# Silkworm Storage Protein 1 Inhibits Autophagy-Mediated Apoptosis

**DOI:** 10.3390/ijms20020318

**Published:** 2019-01-14

**Authors:** Su Jin Kang, Won Jong Rhee

**Affiliations:** Division of Bioengineering, Incheon National University, 119 Academy-ro, Yeonsu-gu, Incheon 406-772, Korea; sjkang2525@gmail.com

**Keywords:** autophagy, apoptosis, silkworm, storage protein 1

## Abstract

Autophagy is a natural physiological process, and it induces the lysosomal degradation of intracellular components in response to environmental stresses, including nutrient starvation. Although an adequate autophagy level helps in cell survival, excessive autophagy triggered by stress such as starvation leads to autophagy-mediated apoptosis. Chinese hamster ovary (CHO) cells are widely used for producing biopharmaceuticals, including monoclonal antibodies. However, apoptosis induced by high stress levels, including nutrient deficiency, is a major problem in cell cultures grown in bioreactors, which should be overcome. Therefore, it is necessary to develop a method for suppressing excessive autophagy and for maintaining an appropriate autophagy level in cells. Therefore, we investigated the effect of silkworm storage protein 1 (SP1), an antiapoptotic protein, on autophagy-mediated apoptosis. SP1-expressing CHO cells were generated to assess the effect and molecular mechanism of SP1 in suppressing autophagy. These cells were cultured under starvation conditions by treatment with Earle’s balanced salt solution (EBSS) to induce autophagy. We observed that SP1 significantly inhibited autophagy-mediated apoptosis by suppressing caspase-3 activation and reactive oxygen species generation. In addition, SP1 suppressed EBSS-induced conversion of LC3-I to LC3-II and the expression of autophagy-related protein 7. Notably, basal Beclin-1 level was significantly low in the SP1-expressing cells, indicating that SP1 regulated upstream events in the autophagy pathway. Together, these findings suggest that SP1 offers a new strategy for overcoming severe autophagy-mediated apoptosis in mammalian cells, and it can be used widely in biopharmaceutical production.

## 1. Introduction

Autophagy is the process of programmed cell death II that may occur as a part of bioenergetic control that is initiated by environmental stresses such as nutrient starvation, and osmotic and oxidative stresses [1,2,3]. It is a homeostatic self-cannibalization process that involves the degradation of cellular components, and removal of damaged organelles. During autophagy, intracellular components are enclosed within a double-membrane vesicle called autophagosome [4]. Unc-51-like kinase (ULK), which forms a complex with FIP200/ATG13, acts on the multiple steps of the autophagy pathway and phosphorylates autophagy-related proteins (ATGs), including Beclin-1 [5]. ULK kinase activity is regulated by phosphorylation mediated by the mammalian target of rapamycin (mTOR), which senses nutrients and stress [6,7]. Although mTOR suppresses the ULK complex under nutrient-rich conditions, it activates this complex under starvation, which induces Beclin-1 to promote phagophore formation [8,9,10]. The ATG system then promotes the conversion of microtubule-associated protein light chain 3 (LC3), which is involved in the initiation of autophagosome formation. After autophagy initiation, pro-LC3 is cleaved at its C-terminus to produce LC3-I. The soluble cytoplasmic LC3-I binds to phosphatidylethanolamine to produce LC3-II, which then binds to both the inner and outer autophagosome membranes to complete autophagosome formation. Autophagosomes then fuse with lysosomes to form autolysosomes, which decompose the enclosed intracellular components [11].

Interestingly, although the basic autophagy level helps in cell survival, excessive autophagy induced by stress, including starvation, induces apoptosis [12]. Thus, suppression of excessive autophagy can reduce starvation-induced apoptosis [13,14,15,16,17]. Moreover, the maintenance of an appropriate autophagy level is required in major human diseases such as neurological diseases, cancers, cardiovascular diseases, diabetes, and infection-related diseases [18,19,20]. In addition, it is necessary to inhibit severe autophagy-mediated apoptosis induced by starvation in a bioreactor during the production of biopharmaceutical drugs. Chinese hamster ovary (CHO) cells are widely used for producing biopharmaceutical drugs [21,22,23,24]. High cell density and prolonged cell culture duration are the key factors required for achieving high productivity and product quality. During cell culture in a bioreactor, cells are exposed to severe metabolic stress caused by nutrient depletion or oxidative stress, especially in the late culture stage. This severe metabolic stress triggers autophagy followed by apoptosis, thus decreasing cell concentration and cellular activities for drug production [25,26,27]. Therefore, it is important to develop a method for protecting CHO cells from autophagy-mediated apoptosis during culture.

Silkworm storage protein 1 (SP1) functions as hemoglobin in the silkworm *Bombyx mori*, and it is a major component of its hemolymph. SP1 contains an α-helical domain, a copper-binding domain, and an Ig-like domain. Previous studies have reported the inhibitory effects of SP1 on apoptosis induced by staurosporine (STS) through an intrinsic mitochondria-mediated pathway and on oxidative stress; however, the effects of SP1 on autophagy have not been reported to date [28,29,30]. Because autophagy-mediated apoptosis is a critical problem in the bioindustry, and in many diseases, it is important to determine the effect of SP1 on starvation-induced autophagy and apoptosis.

In the present study, we investigated the effect and molecular mechanism of SP1 in inhibiting starvation-induced apoptosis in CHO cells. Starvation-induced autophagy in CHO cells was elicited using Earle’s balanced salt solution (EBSS). Stable SP1-expressing CHO and HeLa cells (CHO/SP1 and HeLa/SP1 cells, respectively) were generated to evaluate the biological role of SP1 in autophagy. Intracellular levels of Beclin-1, autophagy-related protein 7 (ATG7), LC3-I, and LC3-II were analyzed to assess the effect of SP1 on starvation-induced autophagy. Moreover, cell viability and reactive oxygen species (ROS) generation were analyzed to assess the effects of SP1 on severe autophagy-mediated apoptosis.

## 2. Results

### 2.1. SP1 Inhibits Cell Death Induced by EBSS Treatment

**Figure 1:**
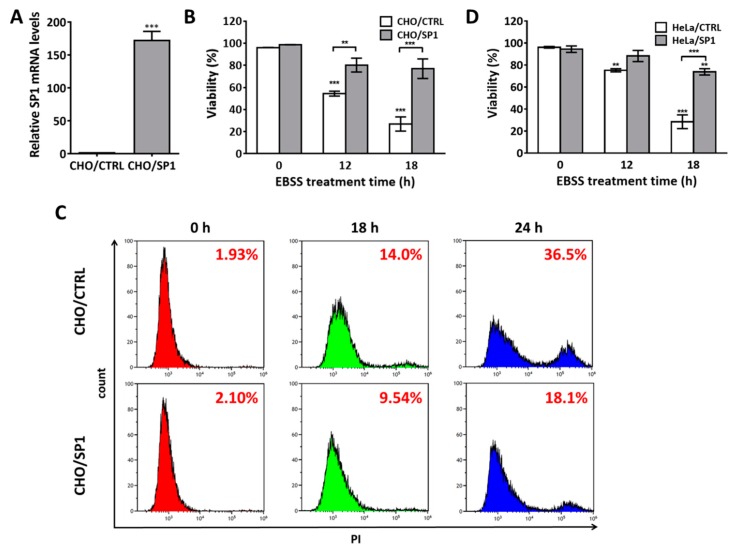
SP1 inhibits autophagy-mediated cell death induced by Earle’s balanced salt solution (EBSS) treatment. (**A**) The expression of SP1 messenger RNA (mRNA) in each cell line was confirmed by reverse transcriptase polymerase chain reaction (PCR) analysis. (**B**) The effect of SP1 on cell death in the Chinese hamster ovary (CHO) cells treated with EBSS for 0, 12, and 18 h. Cell viability was measured by performing the Trypan blue dye exclusion assay. (**C**) Flow cytometry analysis of cell death before and after the EBSS treatment in the CHO/CTRL and CHO/SP1 cells. The cells were treated with EBSS for 0, 18, and 24 h, and were stained with PI before performing the flow cytometry analysis. (**D**) The effect of SP1 on autophagy-mediated cell death in the HeLa cells treated with EBSS. All values are represented as mean ± SD (** *p* < 0.01 and *** *p* < 0.001; *n* = 3).

EBSS is widely used for inducing autophagy in mammalian cells, and prolonged exposure of cells to EBSS induces apoptosis [31,32,33,34,35]. The effects of SP1 on EBSS-induced autophagy and autophagy-mediated apoptosis were evaluated using the stable SP1-expressing CHO cells (CHO/SP1 cells). The CHO cells not expressing SP1 (CHO/CTRL cells) were used as the control cells. SP1 expression was confirmed by reverse transcriptase polymer chain reaction (PCR), as shown in Figure 1A. Cell viabilities were determined by performing the Trypan blue dye exclusion assay before and after the EBSS treatment. EBSS treatment decreased the viability of the CHO/CTRL cells in a time-dependent manner, with the percentage of viable CHO/CTRL cells decreasing to 54.8% and 26.9% after EBSS treatment for 12 and 18 h, respectively (Figure 1B). In contrast, EBSS treatment decreased the percentage of viable CHO/SP1 cells to 80.3% and 76.4% after 12 and 18 h, respectively, indicating that SP1 inhibited autophagy-mediated apoptosis.

We further assessed cell viability by performing flow cytometry analysis to determine the effect of SP1 on autophagy-mediated apoptosis (Figure 1C). The cells were treated with EBSS for 18 and 24 h and were stained with PI to assess their viability. EBSS treatment for 18 and 24 h decreased the viability of the CHO/CTRL cells by 14.0% and 36.5%, respectively, and that of the CHO/SP1 by only 9.54% and 18.1%, respectively, indicating that SP1 expression increased the resistance of CHO cells to autophagy-mediated apoptosis.

To rule out the possibility that SP1-induced inhibition of EBSS-associated apoptosis was limited to CHO cells, HeLa cells expressing and not expressing SP1 (HeLa/SP1 and HeLa/CTRL, respectively) were treated with EBSS for 12 and 18 h, and the effect of SP1 expression on apoptosis inhibition was determined. EBSS treatment for 18 h drastically decreased the percentage of viable HeLa/CTRL cells to 28.5%, but decreased the percentage of viable HeLa/SP1 cells to 73.8% (Figure 1D). This result indicates that SP1-induced inhibition of EBSS-induced apoptosis is not limited to CHO cells. Overall, these results clearly suggest that SP1 inhibits autophagy-mediated apoptosis.

### 2.2. Effects of SP1 Expression on Caspase-3 Activation and ROS Generation

**Figure 2:**
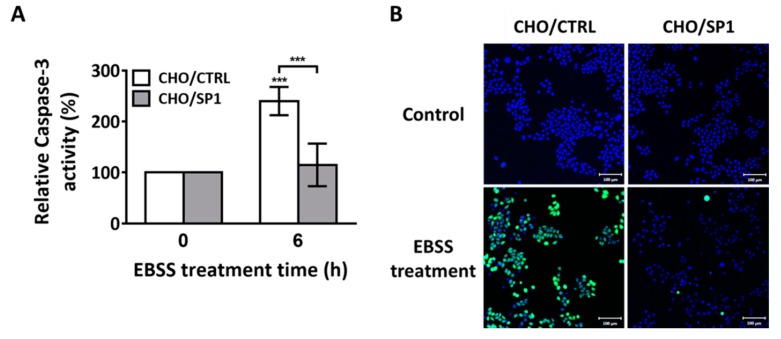
Effects of SP1 on caspase-3 activation and reactive oxygen species (ROS) generation in the EBSS-treated CHO cells. (**A**) The effect of SP1 on caspase-3 activity after autophagy induction. The cells were treated with EBSS for 6 h, and caspase-3 activity was assessed using the caspase-3 substrate N-Acetyl-Asp-Glu-Val-Asp-7-amido-4-Trifluoromethylcoumarin (Ac-DEVD-AFC). (**B**) The effect of SP1 on the ROS generation in cells under starvation. ROS levels were measured using the cell permeant 2′,7′-dichlorodihydrofluorescein diacetate (H_2_DCFDA), and were analyzed by performing fluorescence microscopy. The cells were treated with EBSS for 0 and 4 h, and were stained with H_2_DCFDA (green) for measuring intracellular ROS levels and with Hoechst 33342 (blue) for staining the nucleus. All values are represented as mean ± SD (*** *p* < 0.001; *n* = 3).

We further investigated the effect of SP1 expression on caspase-3 activation, a downstream event in apoptosis after EBSS treatment. The cells were cultured and exposed to EBSS for 6 h, and caspase-3 activity was measured using cell lysates. Caspase-3 activity increased to 240% in the CHO/CTRL cells but was significantly suppressed (only 115%) in the CHO/SP1 cells (Figure 2A). These results indicate that SP1 expression protects CHO cells from starvation-induced apoptosis associated with the EBSS treatment by suppressing caspase-3 activation.

During apoptosis, ROS generation is the key event that occurs upstream of caspase-3 activation [3,36,37,38,39,40]. SP1 exerts strong antioxidant effects in cells exposed to oxidative stress. To explore whether SP1 expression inhibited ROS generation in CHO cells, the CHO/CTRL and CHO/SP1 cells were starved by treatment with EBSS for 4 h, and were stained with the H_2_DCFDA dye to measure intracellular ROS levels. EBSS treatment drastically increased intracellular ROS levels in the CHO/CTRL cells (indicated by strong fluorescence signals) but it negligibly increased intracellular ROS levels in the CHO/SP1 cells (indicated by almost negligible fluorescence signals) (Figure 2B). These results indicate that SP1 expression inhibits ROS generation during starvation-induced autophagy.

### 2.3. SP1 Inhibits LC3 Conversion

**Figure 3:**
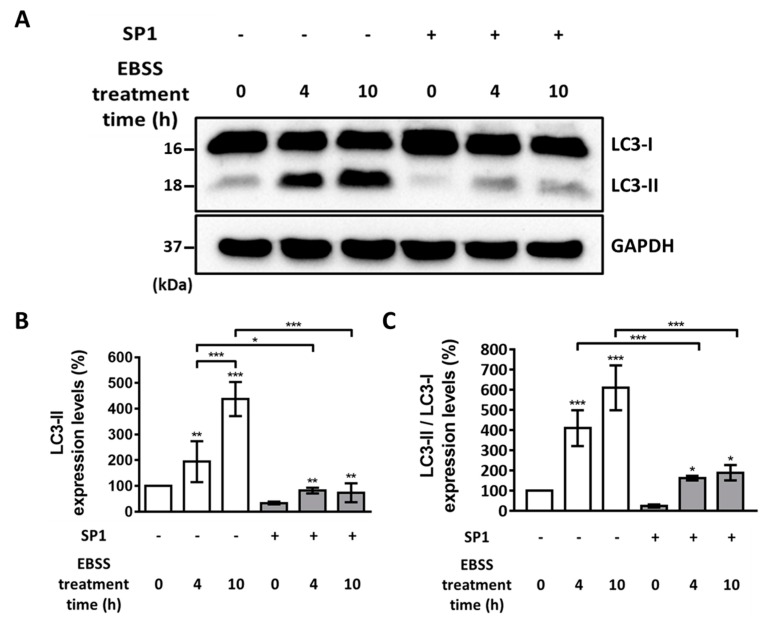
Western blotting analysis of LC3-I and LC3-II levels in the CHO cells before and after the EBSS treatment. (**A**) LC3-I and LC3-II protein levels were analyzed by performing Western blotting analysis. The cells were treated with EBSS for 0, 4, and 10 h. Glyceraldehyde 3-phosphate dehydrogenase (GAPDH) was used as an internal control. (**B**) Quantitative analysis of the results of Western blotting analysis are shown in (**A**) for LC3-II levels normalized using the GAPDH levels. Statistical analysis of data obtained from three independent experiments was performed using GraphPad Prism 7.0 software and ImageJ 1.43. (**C**) Quantitative analysis of the results of Western blot analysis shown in (A) for LC3-II levels normalized using LC3-I levels. All values are represented as mean ± SD (* *p* < 0.05, ** *p* < 0.01, and *** *p* < 0.001; *n* = 3).

Based on the above results, we hypothesized that EBSS-treated cells showed autophagy induction in response to starvation. The accumulation of cellular stress triggers apoptotic signals. Because SP1 inhibited starvation-induced apoptosis, it is plausible that it inhibits the conversion of soluble LC3-I to lipid-bound LC3-II during autophagy [17,41,42,43,44]. Therefore, we determined cellular LC3-I and LC3–II levels by performing Western blotting analysis before and after EBSS treatment in each cell line (Figure 3). Treatment of the CHO/CTRL cells with EBSS decreased LC3-I levels and drastically increased LC3-II levels in a time-dependent manner (Figure 3A). This result indicates that starvation induces an autophagy signaling cascade in cells, and it results in the conversion of a significant amount of LC3-I to LC3-II. In contrast, a limited increase in LC3-II level was observed in the EBSS-treated CHO/SP1 cells. The results of Western blotting analysis for LC3–II were quantitatively analyzed, using GAPDH as the normalizing protein (Figure 3B). These results are expressed as the means of relationships between the densitometries of LC3-II and GAPDH. In the CHO/CTRL cells, LC3-II level significantly increased to 194% and 438% after EBSS treatment for 4 and 10 h, respectively. In contrast, in the CHO/SP1 cells, LC3-II level increased by only 82% and 73% after EBSS treatment for 4 and 10 h, respectively, indicating that SP1 inhibited the conversion of LC3-I to LC3-II in SP1-expressing CHO cells. Next, we assessed the ratio of LC3-II to LC3-I by using the results of the Western blotting analysis. We observed that this ratio significantly increased to 409% and 609% in the CHO/CTRL treated with EBSS for 4 and 10 h, respectively (Figure 3C). In contrast, the fold-increase in the ratio of LC3-II to LC3-I was clearly reduced in the CHO/SP1 cells (162% and 188% after EBSS treatment for 4 and 10 h, respectively). Overall, these results strongly indicate that SP1 expression blocks autophagy-mediated apoptosis by inhibiting the conversion of LC3-I to LC3-II, and thus, by delaying the autophagosome formation in starving cells.

### 2.4. SP1 Regulates ATG7 Expression Level

**Figure 4:**
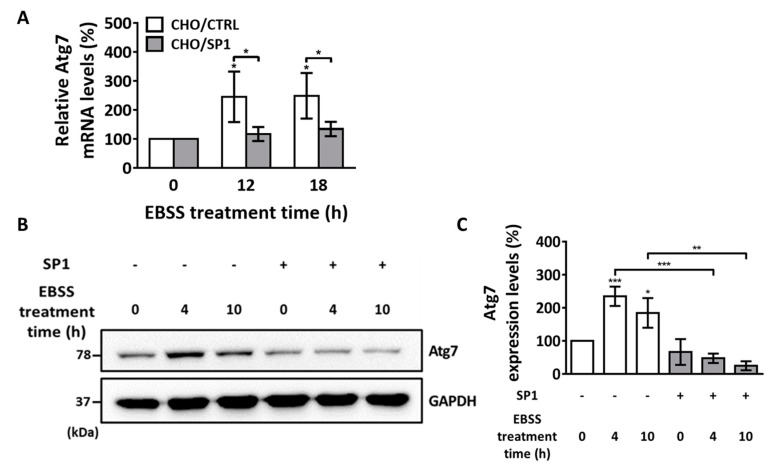
The mRNA and protein levels of the key regulator ATG7 during EBSS-induced autophagy. (**A**) Quantitative PCR analysis of ATG7 mRNA levels in the CHO/CTRL and CHO/SP1 cells treated with EBSS for 0, 12, and 18 h. (**B**) Western blotting analysis of ATG7 protein levels in the CHO cells treated with EBSS for 0, 4, and 10 h. GAPDH was used as an internal control (**C**) Quantitative analysis of the results of Western blot analysis shown in (**B**) for ATG7 levels normalized using the GAPDH levels. Statistical analysis of data obtained from three independent experiments was performed using GraphPad Prism 7.0 software and ImageJ 1.43. All values are represented as mean ± SD (* *p* < 0.05, ** *p* < 0.01, and *** *p* < 0.001; *n* = 3).

To explore the molecular mechanism underlying SP1-induced inhibition of autophagy-mediated apoptosis, we assessed the effect of SP1 on ATG7 expression after EBSS treatment. Moreover, we investigated whether ATG7 mediated the inhibitory effect of SP1 on autophagy-mediated apoptosis (Figure 4). Mammalian ATG7 is essential for the ATG conjugation system and for autophagosome formation. ATG7 regulates the conversion of LC3-I to LC3-II [45,46,47,48]. Because SP1 suppressed the conversion of LC3-I to LC3-II (Figure 3A), we hypothesized that SP1 also suppressed EBSS-induced increase in ATG7 expression level. To assess this, both the CHO/SP1 and CHO/CTRL cells were treated with EBSS for 12 and 18 h. In the CHO/CTRL cells, EBSS treatment drastically increased ATG7 messenger RNA (mRNA) expression at 12 hr; moreover, this increase in the ATG7 mRNA expression level was maintained at 18 h after the EBSS treatment (Figure 4A). Furthermore, the fold-increase in the ATG7 mRNA expression was 246% and 249% after EBSS treatment for 12 and 18 h, respectively, in the CHO/CTRL cells. In contrast, no significant increase in the ATG7 mRNA expression was observed in the EBSS-treated CHO/SP1 cells. The relative ATG7 mRNA expression levels were 117% and 135% after EBSS treatment for 12 and 18 h, respectively, in the CHO/SP1 cells. Western blotting analysis was performed to verify whether SP1 suppressed ATG7 protein expression under starvation. No significant difference in the basal ATG7 expression level was observed between the CHO/CTRL and CHO/SP1 cells before the EBSS treatment (Figure 4B,C). However, ATG7 expression level increased to 357% and 280% at 4 and 10 h, respectively, in the EBSS-treated CHO/CTRL cells, whereas no recognizable change in the ATG7 expression level was observed in the EBSS-treated CHO/SP1 cells. These results indicate that SP1 inhibits the increase in LC3-II formation by suppressing ATG7 expression level during starvation-induced autophagy, thus subsequently inhibiting apoptosis.

### 2.5. Effect of SP1 on Beclin-1 Expression

**Figure 5:**
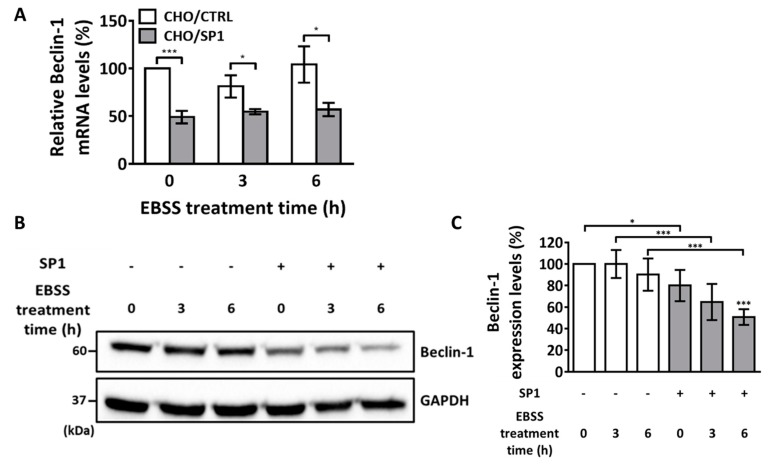
The mRNA and protein levels of Beclin-1 after the EBSS treatment. (**A**) Quantitative PCR analysis of Beclin-1 mRNA levels in the CHO/CTRL and CHO/SP1 cells treated with EBSS for 0, 12, and 18 h. (**B**) Western blotting analysis of Beclin-1 protein levels in the CHO cells treated with EBSS for 0, 4, and 10 h. GAPDH was used as an internal control. (**C**) Quantitative analysis of the results of Western blotting analysis shown in (**B**) for Beclin-1 levels normalized using the GAPDH levels. Statistical analysis of data obtained from three independent experiments was performed using GraphPad Prism 7.0 software and ImageJ 1.43. All values are represented as mean ± SD (* *p* < 0.05, ** *p* < 0.01, and *** *p* < 0.001; *n* = 3).

Beclin-1, which is core component of a type III phosphatidylinositol 3-kinase complex is a part of a protein complex confined to the autophagosome membrane [49]. Beclin-1 is the key regulator of autophagy and contributes to autophagosome formation; moreover, low Beclin-1 expression in cells inhibits autophagy [50]. Thus, Beclin-1 is an upstream marker for autophagy induction in mammalian cells. In the present study, Beclin-1 mRNA and protein levels before and after the EBSS treatment were measured by performing real-time PCR and Western blotting analysis, respectively (Figure 5). Although Beclin-1 level increases after EBSS treatment, Beclin-1 mRNA level did not increase in both the CHO/CTRL and CHO/SP1 cells after EBSS treatment in the present study (Figure 5A). In the CHO/CTRL cells, the fold increase in Beclin-1 mRNA level was 81% and 104% after EBSS treatment for 12 and 18 h, respectively, indicating that the EBSS treatment did not affect Beclin-1 mRNA level. Similar results were obtained for the CHO/SP1 cells, with the Beclin-1 mRNA level being increased by 55% and 57% after EBSS treatment for 12 and 18 h, respectively. Results of the Western blotting analysis of Beclin-1 protein levels in both the CHO/CTRL and CHO/SP1 cells confirmed the results of the real-time PCR analysis that the EBSS treatment did not increase Beclin-1 levels in our experimental setting (Figure 5B). Surprisingly, basal Beclin-1 levels was significantly lower in the CHO/SP1 cells than in the CHO/CTRL cells (Figure 5B). Quantitative analysis of the results of the Western blotting analysis showed that the Beclin-1 level before the EBSS treatment was 80% in the CHO/SP1 cells, which was 20% lower than that in the CHO/CTRL cells (Figure 5C). In addition, the Beclin-1 level gradually decreased after the EBSS treatment only in the CHO/SP1 cells. Beclin-1 level in the CHO/SP1 cell was 51% while CHO/CTRL cell was 90%, respectively, at 6 h after the EBSS treatment. These results suggest that SP1 expression significantly affected Beclin-1 level before and after the EBSS treatment, and that this contributes to the inhibition of autophagy progression in CHO cells under starvation.

### 2.6. Effect of SP1 on Autophagy Blockade-Induced Cell Death

**Figure 6:**
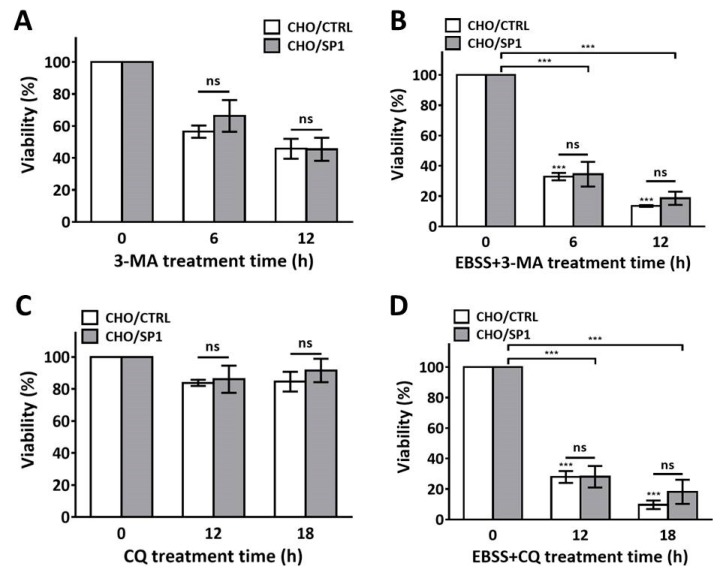
The effect of SP1 on cell viabilities in autophagy blockade. (**A**,**B**) The effect of SP1 on cell death in the CHO cells treated with 3-MA for 0, 6, and 12 h in the (**A**) absence or (**B**) presence of EBSS. (**C**,**D**) The effect of SP1 on cell death in the CHO cells treated with CQ for 0, 12, and 18 h in the (**C**) absence or (**D**) presence of EBSS. Cell viability was measured by performing the Trypan blue dye exclusion assay. All values are represented as mean ± SD (*** *p* < 0.001; *n* = 3).

To investigate SP1 effect on apoptosis in the presence of autophagy inhibitor, cells were treated with 3-,ethyladenine (3-MA) under starvation or non- starvation conditions. First, cells started to die after 3-MA treatment regardless of SP1 expression, even in the absence of EBSS (Figure 6A). Cell viabilities dropped to 42.1% and 49.1% at 12 h after 3-MA treatment in CHO/CTRL and CHO/SP1, respectively. EBSS and 3-MA co-treatment further decreased cell viabilities in both cell lines, and there was no significant difference between CHO/CTRL and CHO/SP1 (Figure 6B). Although 3-MA inhibits autophagy process in cells, it was previously reported that 3-MA treatment itself induced apoptosis without autophagy [51,52]. The entire blockade of autophagy in cells rather caused apoptosis, even in the absence of starvation. As SP1 did not inhibit apoptosis caused by autophagy blockade, it is obvious that SP1 specifically suppresses starvation-induced autophagy-mediated apoptosis and cannot suppress starvation-induced apoptosis in the absence of autophagy. In addition, cells were also treated with chloroquine (CQ), the downstream autophagy inhibitor that causes autophagosome accumulations, under starvation or non- starvation conditions, to see the SP1 effect on cell viability. As shown in Figure 6C, CQ treatment itself did not cause apoptosis and no significant decrease in cell viabilities was observed in both cell lines. In addition, EBSS and CQ co-treatment drastically decreased the cell viabilities regardless of SP1 expression (Figure 6D). These phenomena were previously reported by other groups that CQ treatment under starvation condition induced cell death, although CQ itself did not induce cell death, including both apoptosis and necrosis [40]. As there was no significant difference in cell viabilities between CHO/CTRL and CHO/SP1 after CQ treatment under starvation condition, it is speculated that CQ and EBSS co-treatment may induce cell death that has a different molecular mechanism as compared to autophagy-mediated apoptosis induced by EBSS treatment. In addition, it is also plausible that SP1 can specifically inhibit apoptosis when the normal autophagic flux is present.

## 3. Discussion

**Figure 7:**
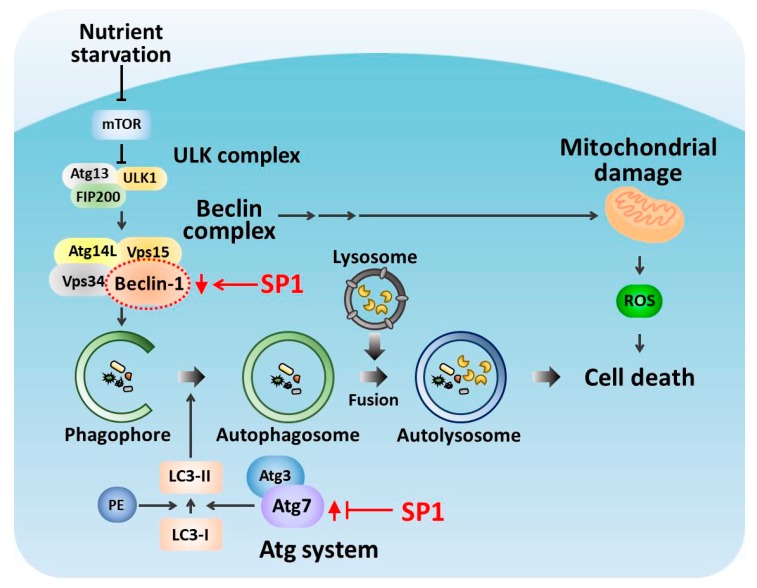
The predicted molecular pathway of SP1 involved in the inhibition of autophagy-mediated apoptosis induced by starvation. SP1 suppresses the EBSS-induced increase in ATG7 level, and regulates the basal Beclin-1 level.

SP1 suppresses apoptosis induced by a broad range of apoptotic inducers, including STS (a mitochondria-mediated apoptosis inducer), hydrogen peroxide (an oxidative stress-mediated apoptosis inducer), and thapsigargin (an endoplasmic reticulum (ER)-mediated apoptosis inducer) [28,29,30,53]. Interestingly, SP1 does not directly inhibit caspases, including caspase-3, which are the downstream mediators of apoptosis. Instead, SP1 functions as the upstream inhibitor of apoptosis by protecting the mitochondria or by suppressing calcium release from the ER into the cytosol. Therefore, SP1 may be a multi-functional protein acting on different sites in molecular pathways controlling apoptosis. In the present study, SP1 suppressed EBSS-induced conversion of LC3-I to LC3-II (Figure 3). The LC3-II formation blockage under starvation condition may subsequently inhibit the autophagosome formation in the cells. As there will be a lesser amount of autolysosome formed, this will finally end up with reduce apoptosis level. The LC3-I conversion to LC3-II is regulated by the ATG system, including the ATG7 level during starvation. As ATG7 expression level was significantly suppressed by SP1 expression in cells (Figure 4), this explains how the LC3-II formation followed by apoptosis induction was suppressed by SP1 (Figure 7). Moreover, the basal Beclin-1 level was low in the SP1-expressing cells and reduced further after the EBSS treatment. This finding suggests that decreased Beclin-1 level in the SP1-expressing cells reduces mitochondrial damage, and in turn, ROS generation. Because increased ROS levels trigger apoptosis cascades, reduction of ROS production by SP1 under starvation may suppress cell death (Figure 7). Thus, these dual effects of SP1 (ATG7 suppression, low Beclin-1 basal level) on autophagy synergistically alleviate intracellular stress, increase the resistance of cells to starvation, and suppress autophagy-mediated apoptosis. It will be very interesting to figure out how SP1 exerts its effect on ATG7 and Beclin-1. As their expression levels in the cells were affected by SP1, it is speculated that SP1 regulates upstream events in autophagy progression, rather than directly interacting with those proteins. Although the molecular mechanism of the connection between Beclin-1 and mitochondrial damage is not fully demonstrated, it seems that Beclin-1 itself does not directly regulate mitochondria when cells are exposed to starvation. Rather, it was reported that Bcl-2, an anti-apoptotic protein that protects mitochondria, negatively regulates the Beclin complex by binding directly to Beclin-1 on the endoplasmic reticulum to inhibit autophagy-mediated cell death, under starvation conditions [54]. Less autophagy and autophagy-mediated apoptosis occurs when Beclin-1 binds to Bcl-2. Unlike Bcl-2, SP1 down-regulated Beclin-1 expression level such that there were fewer starvation-induced autophagy signals in the cells, and this subsequently contributed to apoptosis inhibition. Further studies are required to investigate the exact molecular mechanism of this interesting and multi-functional silkworm protein in cells undergoing autophagy-mediated apoptosis. 

In mammalian cells, excessive stress, including starvation, can induce severe autophagy and eventually apoptosis. Suppression of severe autophagy-mediated apoptosis is required in CHO cells used for producing biopharmaceuticals, because these cells are exposed to stressful conditions, including starvation, in a bioreactor. In addition, several human diseases, including cancer and cardiovascular diseases, are closely associated with severe autophagy; therefore, alleviation and maintenance of an appropriate autophagy level is important for preventing cell death. Different strategies can be adopted for the SP1 applications. For the SP1 application on biopharmaceutical production in the bioreactor, the SP1 coding gene can be cloned into the production cell line to endow it with the resistance to autophagy-mediated apoptosis. On the contrary, to investigate the in vivo effect of SP1 on autophagy-associated diseases, SP1 is required to be produced as a recombinant protein for in vivo administration. In our previous study, recombinant SP1 also showed the anti-apoptosis effect when supplemented to cells. As SP1 is a cell-penetrating protein, recombinant SP1 can be effectively delivered into cells, and it exerts its resistance to autophagy-mediated apoptosis. Based on this, further studies are required to explore the in vivo effects of SP1 on diseases that are closely associated autophagy-mediated apoptosis.

The present study is the first to show that SP1 functions as an inhibitor of autophagy-mediated apoptosis and an upstream regulator of autophagy by suppressing Beclin-1 and ATG7 expression. Thus, the results of the present study suggest that SP1 offers a new strategy to overcome severe autophagy-mediated apoptosis in mammalian cells, and it can be widely used in the medicine and biopharmaceutical industries. 

## 4. Materials and Methods 

### 4.1. Cell Culture and Autophagy Induction

This study used two stable CHO-K1 cell lines, CHO/CTRL and CHO/SP1 (generated by transfecting the CHO-K1 cells with pcDNA3.1-CTRL and pcDNA3.1-SP1, respectively) and two stable HeLa cell lines HeLa/CTRL and HeLa/SP1 (generated by transfecting the HeLa cells with pcDNA3.1-CTRL and pcDNA3.1-SP1, respectively). The cells were grown in Iscove’s modified Dulbecco’s medium (IMDM; Thermo Scientific, Waltham, MA, USA) supplemented with 10% fetal bovine serum (Young In Frontier, Seoul, Korea) and 1% penicillin and streptomycin (Life Technologies, Carlsbad, CA, USA). G418 (Geneticin; Duchefa Biochemie, Haarlem, Netherlands) was added to the IMDM to achieve a final concentration to 500 μg/mL. The cells were maintained in 25-cm^2^ T-flasks and were incubated at 37°C in an atmosphere of 5% CO_2_ to produce monolayer cultures. Starvation was induced by incubating the cells with EBSS (Life Technologies). For autophagy inhibition, 5 mM of 3-Methyladenine (3-MA; Sigma, St. Louis, MO, USA) or 20 μΜ of chloroquine diphosphate (CQ; Sigma) were treated in the absence or presence of EBSS.

### 4.2. Cell Viability Assay

The cells were seeded (density, 5 × 10^4^ cells/cm^2^) into a 24-well plate for cell counting. The autophagy inducer EBSS was added to the culture medium on day 2 of the culture, and the cells were incubated for 12 and 18 h. The number of viable cells was determined by performing a Trypan blue dye exclusion assay with a hemocytometer.

For performing flow cytometry analysis, the cells were collected through centrifugation at 1500 rpm for 5 min, washed twice with phosphate-buffered saline (PBS), stained with 10 µg/mL propidium iodide (PI; Thermo Scientific) dissolved in 100 µL PBS, and incubated for 30 min. Next, the cells were transferred to round-bottom tubes, followed by the addition of 400 µL PBS. The cells were analyzed using a flow cytometer (Beckman Coulter, Miami, FL, USA), which counted at least 10,000 cells per sample. The fraction of cells in each quadrant was calculated using Kaluza software (Beckman Coulter).

### 4.3. Relative Caspase-3 Activity Measurement

Caspase-3 activity was measured by using EBSS-treated stable CHO/CTRL and CHO/SP1 cells. The cells were harvested, washed twice with cold PBS, and lysed with 200 μL lysis buffer (prepared by adding 10 µL protease inhibitors (Thermo Scientific) to 990 µL radioimmunoprecipitation assay (RIPA) buffer (ELPIS-Biotech, Daejeon, Korea)). Protein quantification was performed using bicinchoninic acid (BCA) assay. For this, the cell lysates were diluted and transferred to black 384-well plates (Sigma, St. Louis, MO, USA), followed by the addition of an equal volume of 1× reaction buffer (Ac-DEVD-AFC substrate (Enzo, USA) dissolved in dimethyl sulfoxide (DMSO) and added to 1× 4-(2-hydroxyethly)-1-piperazineethanesulfonic acid (HEPES) buffer) to the wells. After incubation for 1 h at 37°C, fluorescence intensity was measured using a Varioskan™ Flash Multimode Reader (Thermo Scientific) at emission and excitation wavelengths of 400 and 505 nm, respectively.

### 4.4. Intracellular ROS Level Measurement

Intracellular ROS levels were measured by incubating the EBSS-treated stable CHO/CTRL and CHO/SP1 cells with 10 μM of the cell permeant 2′,7′-dichlorodihydrofluorescein diacetate (H_2_DCFDA; Thermo Scientific) for 1 h at 37°C. After incubation, the cells were treated with a fluorescent dye Hoechst 33342 (Cell Signaling Technology, Danvers, MA, USA) for nuclear staining. Next, the cells were washed twice with PBS, and positively stained cells were determined using a fluorescent microscope (Nikon Corp., Minato, Japan).

### 4.5. Real-Time PCR

Relative abundances of SP1, Beclin-1, and ATG7 mRNA levels was assessed using SYBR Green (Qiagen, Venlo, Netherlands) and the StepOnePlus Real-Time PCR System (Applied Biosystems, , Forster City, CA, USA). The sequences of the primers used for performing the real-time PCR are listed in Table 1. Total RNA was extracted from the two stable CHO-K1 cell lines by using Tris-RNA reagent (Favorgen, Taiwan), according to the manufacturer’s instructions. Next, complementary DNA (cDNA) was synthesized using 100 ng total RNA and ReverTra Ace qPCR RT Master Mix (Toyobo, Osaka, Japan). PCR was performed using the following cycling conditions: initial denaturation at 95 °C for 5 min, followed by 40 cycles of denaturation at 95 °C for 30 s, annealing at 60 °C for 30 s, and extension at 72 °C for 1 min. Relative mRNA expression of the target genes was calculated using the comparative Δ*C*t method. All data were controlled for the quantity of RNA input by using the endogenous β-actin gene as the reference.

### 4.6. Western Blotting Analysis

The CHO cells were washed with ice-cold PBS and lysed by using a lysis buffer (ELPIS-Biotech) with a scraper. Total proteins present in the cell lysates were quantified using the BCA assay, according to the manufacturer’s instructions. Next, the cell lysates were added to 5× sodium dodecyl sulfate (SDS) sample buffer and were boiled at 95 °C for 5 min. Proteins present in the cell lysates were resolved by performing SDS-polyacrylamide gel electrophoresis on a 12% gel by loading 20 µg cell lysates. The resolved proteins were transferred onto a nitrocellulose membrane (Bio-Rad, Hercules, CA, USA). Non-specific binding was blocked by incubating the membrane with 5% skimmed milk (BD Biosciences, San Jose, CA, USA) in TBS containing 0.1% Tween 20 (TBS-T) for 1 h. Next, the membrane was incubated overnight at 4°C with the following primary antibodies: anti-LC3 antibody (Sigma), anti-Beclin-1 antibody (Cell Signaling Technology), anti-ATG7 antibody (Cell Signaling Technology), and anti-GAPDH antibody (Sigma). Next, the membrane was washed with TBS-T, followed by incubation with horseradish peroxidase (HRP)-conjugated anti-rabbit IgG secondary antibodies (Cell Signaling Technology) for 1 h at room temperature. The membrane was washed again with TBS-T, and the secondary antibodies were detected using ECL (GE Healthcare, Chicago, IL, USA, UK). Protein bands were visualized using ChemiDoc™ XRS+ System (Bio-Rad) and Image Lab software.

### 4.7. Statistical Analysis

Results are expressed as the mean ± standard deviation (SD), *n* ≥ 3. Data were analyzed using one-way and two-way ANOVA, and *p* < 0.05 was considered to be statistically significant. Statistical analysis of Western blotting analysis data obtained from three independent experiments was performed using GraphPad Prism 7.0 software (GraphPad, San Diego, CA, USA) and ImageJ 1.43.

## Figures and Tables

**Table 1:** Primer sequences.

CHO gene	Quantitative real-time polymerase chain reaction (qRT-PCR) primers (5′-3′)
SP1	Forward: ACA TGA AGA TGA AGG AGC TTT GC
	Reverse: TAG AGA CCC TTG CAG TCG GTT
Beclin-1	Forward: CGA ATG TCA GAA CTA CAA ACG
	Reverse: CAG CCT CTC CTC CTC TAA G
Atg7	Forward: GGG AGA AGA ACC AGA AAG G
	Reverse: GAC CAA TCG CCA ACA CAT
β-actin	Forward: AGC TGA GAG GGA AAT TGT GCG
	Reverse: GCA ACG GAA CCG CTC ATT

## References

[1] Han Y.K., Kim Y.G., Kim J.Y., Lee G.M. (2010). Hyperosmotic stress induces autophagy and apoptosis in recombinant Chinese hamster ovary cell culture. Biotechnol. Bioeng..

[2] Mizushima N., Komatsu M. (2011). Autophagy: Renovation of cells and tissues. Cell.

[3] Chen Y., McMillan-Ward E., Kong J., Israels S.J., Gibson S.B. (2008). Oxidative stress induces autophagic cell death independent of apoptosis in transformed and cancer cells. Cell Death Differ..

[4] Marino G., Niso-Santano M., Baehrecke E.H., Kroemer G. (2014). Self-consumption: The interplay of autophagy and apoptosis. Nat. Rev. Mol. Cell Biol..

[5] Jung C.H., Ro S.H., Cao J., Otto N.M., Kim D.H. (2010). mTOR regulation of autophagy. Febs Lett..

[6] Lee J.S., Lee G.M. (2012). Estimation of autophagy pathway genes for autophagy induction: Overexpression of Atg9A does not induce autophagy in recombinant Chinese hamster ovary cells. Biochem. Eng. J..

[7] Zeng X., Kinsella T.J. (2011). Impact of Autophagy on Chemotherapy and Radiotherapy Mediated Tumor Cytotoxicity: “To Live or not to Live”. Front. Oncol..

[8] Maiuri M.C., Zalckvar E., Kimchi A., Kroemer G. (2007). Self-eating and self-killing: Crosstalk between autophagy and apoptosis. Nat. Rev. Mol. Cell Biol..

[9] Johnson S.C., Rabinovitch P.S., Kaeberlein M. (2013). mTOR is a key modulator of ageing and age-related disease. Nature.

[10] Zhao Y.G., Zhang H. (2016). ULK1 cycling: The ups and downs of the autophagy response. J. Cell Biol..

[11] Yamada E., Singh R. (2012). Mapping autophagy on to your metabolic radar. Diabetes.

[12] Scott R.C., Juhasz G., Neufeld T.P. (2007). Direct induction of autophagy by Atg1 inhibits cell growth and induces apoptotic cell death. Curr. Biol..

[13] Arden N., Betenbaugh M.J. (2004). Life and death in mammalian cell culture: Strategies for apoptosis inhibition. Trends Biotechnol..

[14] Pattingre S., Tassa A., Qu X., Garuti R., Liang X.H., Mizushima N., Packer M., Schneider M.D., Levine B. (2005). Bcl-2 antiapoptotic proteins inhibit Beclin 1-dependent autophagy. Cell.

[15] Suzuki S.W., Onodera J., Ohsumi Y. (2011). Starvation Induced Cell Death in Autophagy-Defective Yeast Mutants Is Caused by Mitochondria Dysfunction. PLoS ONE.

[16] Saiki S., Sasazawa Y., Imamichi Y., Kawajiri S., Fujimaki T., Tanida I., Kobayashi H., Sato F., Sato S., Ishikawa K.-I. (2014). Caffeine induces apoptosis by enhancement of autophagy via PI3K/Akt/mTOR/p70S6K inhibition. Autophagy.

[17] Devrim G., Adi K. (2004). Autophagy as a cell death and tumor suppressor mechanism. Oncogene.

[18] Qin Z.H., Wang Y., Kegel K.B., Kazantsev A., Apostol B.L., Thompson L.M., Yoder J., Aronin N., DiFiglia M. (2003). Autophagy regulates the processing of amino terminal huntingtin fragments. Hum. Mol. Genet..

[19] Kim K.H., Jeong Y.T., Oh H., Kim S.H., Cho J.M., Kim Y.N., Kim S.S., Kim D.H., Hur K.Y., Kim H.K. (2013). Autophagy deficiency leads to protection from obesity and insulin resistance by inducing Fgf21 as a mitokine. Nat. Med..

[20] Ralph A., Nixon A.M.C., Paul M. (2000). Mathews, The Endosomal-Lysosomal System of Neurons in Alzheimer’s Disease Pathogenesis. Neurochem. Res..

[21] Butler M. (2005). Animal cell cultures: Recent achievements and perspectives in the production of biopharmaceuticals. Appl. Microbiol. Biotechnol..

[22] Mohan C., Kim Y.-G., Koo J., Lee G.M. (2008). Assessment of cell engineering strategies for improved therapeutic protein production in CHO cells. Biotechnol. J..

[23] Kim J.Y., Kim Y.G., Lee G.M. (2012). CHO cells in biotechnology for production of recombinant proteins: Current state and further potential. Appl. Microbiol. Biotechnol..

[24] Wurm F.M. (2004). Production of recombinant protein therapeutics in cultivated mammalian cells. Nat. Biotechnol..

[25] Han Y.K., Ha T.K., Lee S.J., Lee J.S., Lee G.M. (2011). Autophagy and apoptosis of recombinant Chinese hamster ovary cells during fed-batch culture: Effect of nutrient supplementation. Biotechnol. Bioeng..

[26] Jardon M.A., Sattha B., Braasch K., Leung A.O., Cote H.C., Butler M., Gorski S.M., Piret J.M. (2012). Inhibition of glutamine-dependent autophagy increases t-PA production in CHO cell fed-batch processes. Biotechnol. Bioeng..

[27] Boya P., Gonzalez-Polo R.A., Casares N., Perfettini J.L., Dessen P., Larochette N., Metivier D., Meley D., Souquere S., Yoshimori T. (2005). Inhibition of macroautophagy triggers apoptosis. Mol. Cell. Biol..

[28] Lee J.H., Park T.H., Rhee W.J. (2015). Inhibition of apoptosis in HeLa cell by silkworm storage protein 1, SP1. Biotechnol. Bioprocess Eng..

[29] Lee J.H., Baik J.E., Rhee W.J. (2017). Anti-oxidative effects of silkworm storage protein 1 in HeLa cell. Process Biochem..

[30] Baik J.E., Rhee W.J. (2018). Anti-apoptotic effects of the alpha-helix domain of silkworm storage protein 1. Biotechnol. Bioprocess Eng..

[31] Xia H.-G., Zhang L., Chen G., Zhang T., Liu J., Jin M., Ma X., Ma D., Yuan J. (2014). Control of basal autophagy by calpain1 mediated cleavage of ATG5. Autophagy.

[32] Kim Y.G., Kim J.Y., Mohan C., Lee G.M. (2009). Effect of Bcl-xL overexpression on apoptosis and autophagy in recombinant Chinese hamster ovary cells under nutrient-deprived condition. Biotechnol. Bioeng..

[33] Hwang S.O., Lee G.M. (2008). Nutrient deprivation induces autophagy as well as apoptosis in Chinese hamster ovary cell culture. Biotechnol. Bioeng..

[34] Hailey D.W., Rambold A.S., Satpute-Krishnan P., Mitra K., Sougrat R., Kim P.K., Lippincott-Schwartz J. (2010). Mitochondria supply membranes for autophagosome biogenesis during starvation. Cell.

[35] Zhang Y., Ren S., Liu Y., Gao K., Liu Z., Zhang Z. (2017). Inhibition of Starvation-Triggered Endoplasmic Reticulum Stress, Autophagy, and Apoptosis in ARPE-19 Cells by Taurine through Modulating the Expression of Calpain-1 and Calpain-2. Int. J. Mol. Sci..

[36] Chen Y., Gibson S.B. (2014). Is mitochondrial generation of reactive oxygen species a trigger for autophagy?. Autophagy.

[37] Scherz-Shouval R., Shvets E., Fass E., Shorer H., Gil L., Elazar Z. (2007). Reactive oxygen species are essential for autophagy and specifically regulate the activity of Atg4. Embo J..

[38] Hariharan N., Zhai P., Sadoshima J. (2011). Oxidative Stress Stimulates Autophagic Flux During Ischemia/Reperfusion. Antioxid. Redox Signal..

[39] Lee J., Giordano S., Zhang J. (2012). Autophagy, mitochondria and oxidative stress: Cross-talk and redox signalling. Biochem. J..

[40] Sun K., Xie X., Liu Y., Han Z., Zhao X., Cai N., Zhang S., Song J., Wei L. (2013). Autophagy lessens ischemic liver injury by reducing oxidative damage. Cell Biosci..

[41] Tanida I., Minematsu-Ikeguchi N., Ueno T., Kominami E. (2005). Lysosomal Turnover, but Not a Cellular Level, of Endogenous LC3 is a Marker for Autophagy. Autophagy.

[42] Martinet W., De Meyer G.R., Andries L., Herman A.G., Kockx M.M. (2006). In situ detection of starvation-induced autophagy. J. Histochem. Cytochem. Off. J. Histochem. Soc..

[43] Kimura S., Fujita N., Noda T., Yoshimori T. (2009). Chapter 1 Monitoring Autophagy in Mammalian Cultured Cells through the Dynamics of LC3. Methods Enzymol..

[44] Jean M., Mulcahy Levy A.T. (2012). Modulation of pediatric brain tumor autophagy and chemosensitivity. J. Neurooncol..

[45] He C., Bartholomew C.R., Zhou W., Klionsky D.J. (2009). Assaying autophagic activity in transgenic GFP-Lc3 and GFP-Gabarap zebrafish embryos. Autophagy.

[46] Kouroku Y., Fujita E., Tanida I., Ueno T., Isoai A., Kumagai H., Ogawa S., Kaufman R.J., Kominami E., Momoi T. (2007). ER stress (PERK/eIF2alpha phosphorylation) mediates the polyglutamine-induced LC3 conversion, an essential step for autophagy formation. Cell Death Differ..

[47] Kabeya Y., Mizushima N., Yamamoto A., Oshitani-Okamoto S., Ohsumi Y., Yoshimori T. (2004). LC3, GABARAP and GATE16 localize to autophagosomal membrane depending on form-II formation. J. Cell Sci..

[48] Komatsu M., Waguri S., Ueno T., Iwata J., Murata S., Tanida I., Ezaki J., Mizushima N., Ohsumi Y., Uchiyama Y. (2005). Impairment of starvation-induced and constitutive autophagy in Atg7-deficient mice. J. Cell Biol..

[49] Matsunaga K., Saitoh T., Tabata K., Omori H., Satoh T., Kurotori N., Maejima I., Shirahama-Noda K., Ichimura T., Isobe T. (2009). Two Beclin 1-binding proteins, Atg14L and Rubicon, reciprocally regulate autophagy at different stages. Nat. Cell Biol..

[50] Li D.D., Wang L.L., Deng R., Tang J., Shen Y., Guo J.F., Wang Y., Xia L.P., Feng G.K., Liu Q.Q. (2009). The pivotal role of c-Jun NH_2_-terminal kinase-mediated Beclin 1 expression during anticancer agents-induced autophagy in cancer cells. Oncogene.

[51] Kim H.S., Tian L., Jung M., Choi S.K., Sun Y., Kim H., Moon W.K. (2015). Downregulation of Choline Kinase-Alpha Enhances Autophagy in Tamoxifen-Resistant Breast Cancer Cells. PLoS ONE.

[52] Wu Y.T., Tan H.L., Shui G., Bauvy C., Huang Q., Wenk M.R., Ong C.N., Codogno P., Shen H.M. (2010). Dual role of 3-methyladenine in modulation of autophagy via different temporal patterns of inhibition on class I and III phosphoinositide 3-kinase. J. Biol. Chem..

[53] Cha Y.J., Baik J.E., Rhee W.J. (2018). Inhibition of Endoplasmic Reticulum Stress-induced Apoptosis by Silkworm Storage Protein 1. Biotechnol. Bioprocess Eng..

[54] Rebecca T., Marquez L.X. (2012). Bcl-2: Beclin 1 complex: Multiple, mechanisms regulating autophagy/apoptosis toggle switch. Am. J. Cancer Res..

